# Virion Structure of Black Queen Cell Virus, a Common Honeybee Pathogen

**DOI:** 10.1128/JVI.02100-16

**Published:** 2017-02-28

**Authors:** Radovan Spurny, Antonín Přidal, Lenka Pálková, Hoa Khanh Tran Kiem, Joachim R. de Miranda, Pavel Plevka

**Affiliations:** a Structural Virology, Central European Institute of Technology, Masaryk University, Brno, Czech Republic; b Department of Zoology, Fishery, Hydrobiology, and Apidology, Faculty of Agronomy, Mendel University in Brno, Brno, Czech Republic; c Department of Ecology, Swedish University of Agricultural Sciences, Uppsala, Sweden; Hudson Institute of Medical Research

**Keywords:** virus, Apis mellifera, honey bee, honeybee, Picornavirales, Dicistroviridae, Cripavirus, Triatovirus, virion, structure, X ray, crystallography, capsid, insect disease, X-ray crystallography

## Abstract

Viral diseases are a major threat to honeybee (Apis mellifera) populations worldwide and therefore an important factor in reliable crop pollination and food security. Black queen cell virus (BQCV) is the etiological agent of a fatal disease of honeybee queen larvae and pupae. The virus belongs to the genus Triatovirus from the family Dicistroviridae, which is part of the order Picornavirales. Here we present a crystal structure of BQCV determined to a resolution of 3.4 Å. The virion is formed by 60 copies of each of the major capsid proteins VP1, VP2, and VP3; however, there is no density corresponding to a 75-residue-long minor capsid protein VP4 encoded by the BQCV genome. We show that the VP4 subunits are present in the crystallized virions that are infectious. This aspect of the BQCV virion is similar to that of the previously characterized triatoma virus and supports the recent establishment of the separate genus Triatovirus within the family Dicistroviridae. The C terminus of VP1 and CD loops of capsid proteins VP1 and VP3 of BQCV form 34-Å-tall finger-like protrusions at the virion surface. The protrusions are larger than those of related dicistroviruses.

**IMPORTANCE** The western honeybee is the most important pollinator of all, and it is required to sustain the agricultural production and biodiversity of wild flowering plants. However, honeybee populations worldwide are suffering from virus infections that cause colony losses. One of the most common, and least known, honeybee pathogens is black queen cell virus (BQCV), which at high titers causes queen larvae and pupae to turn black and die. Here we present the three-dimensional virion structure of BQCV, determined by X-ray crystallography. The structure of BQCV reveals large protrusions on the virion surface. Capsid protein VP1 of BQCV does not contain a hydrophobic pocket. Therefore, the BQCV virion structure provides evidence that capsid-binding antiviral compounds that can prevent the replication of vertebrate picornaviruses may be ineffective against honeybee virus infections.

## INTRODUCTION

The honeybee (Apis mellifera) is found all over the world and plays a vital role in the agricultural industry by providing pollination services for food crops. About 10% of the total economic value of agricultural production depends on insect pollination (1). In addition, it has been shown that the abundance and diversity of wild insect-pollinated plant species declines in areas with reduced populations of honeybees (2, 3). However, the bees suffer from a combination of factors such as environmental stress, parasites, and pathogens, including numerous viruses that result in colony losses (4, 5).

One of the most common and least understood honeybee viruses is black queen cell virus (BQCV). BQCV was first isolated from dead queen larvae and prepupae sealed in queen cells with blackened walls (6, 7). BQCV is one of the most common and abundant honeybee viruses worldwide (8–10). It persists chronically and mostly asymptomatically in bee colonies through social transmission among adults and through vertical transmission from the queen to her offspring and from adults to larvae through glandular secretions, e.g., royal jelly (11). However, at elevated titers, BQCV kills developing queen larvae, whose necrotic remains stain their pupal cells black. The disease is of concern for the honeybee queen-rearing industry, but it only rarely has impact outside this context (12, 13). The incidence of BQCV in Europe and Asia peaks during the swarming season, when queens and drones are reared (14–16). There is evidence that the coinfection of BQCV with Nosema, a fungal intestinal parasite of honeybees, results in increased mortality caused by the virus (17). In addition, sublethal doses of pesticides result in increased BQCV titers and mortality (12, 18). BQCV belongs to the family Dicistroviridae, nonenveloped RNA viruses that infect insects (19). The BQCV host range includes many Apis species, as well as several bumblebee species (20). Several other dicistroviruses infect honeybees and bumblebees, whereas others cause diseases in ants, crickets, flies, and aphids.

The structures of Israeli acute bee paralysis virus (IAPV), triatoma virus (TrV), and cricket paralysis virus (CrPV) from the family Dicistroviridae have been determined previously (21–23). IAPV belongs to the genus Aparavirus and CrPV is part of the genus Cripavirus, whereas TrV and BQCV belong to the recently established genus Triatovirus. Viruses from the family Dicistroviridae have nonenveloped icosahedral capsids that protect linear single-stranded positive-sense RNA genomes 8,500 to 10,200 nucleotides in length (24). The genomes of dicistroviruses include two nonoverlapping open reading frames (ORFs), ORF1 and ORF2, which encode polyproteins containing nonstructural and structural (capsid-forming) subunits, respectively. The polyproteins include proteases that cotranslationally and posttranslationally autocleave the polyproteins to produce functional subunits. The major capsid proteins VP1 to VP3 of dicistroviruses have a jelly roll β-sandwich fold common to capsid proteins of many other viruses and form the capsid shell with pseudo-T= 3 icosahedral symmetry (21–23, 25). Capsid proteins originating from one polyprotein precursor fold into a protomer that contains subunits VP0, VP1, and VP2. By analogy with human picornaviruses, it is assumed that the protomers assemble into pentamers and subsequently together with the RNA genome form immature virions (26–29). The cleavage of VP0, which produces subunits VP4 and VP3, is required for the maturation of infectious virions (22, 23). It has been proposed previously that a conserved Asp-Asp-Phe (DDF) motif, which is part of the VP1 subunit and conserved among dicistroviruses, is involved in the VP0 cleavage (22, 23, 30–32). The VP4 subunits of dicistroviruses are peptides 51 to 75 residues long (21–23). CrPV and IAPV virions contain structured VP4 subunits attached to the inner faces of their capsids (21, 22). In contrast, it has been shown that TrV virions contain VP4 subunits, but the TrV crystal structure did not reveal a resolved electron density belonging to VP4 (23). The release of VP4 subunits from virions has been shown to be associated with genome release in the related picornaviruses (33–37). The VP4 subunits disrupt cellular membranes and thus enable the delivery of picornavirus genomes into the cytoplasm (38).

Here we present the structure of the BQCV virion and show that it contains large finger-like surface protrusions formed by capsid proteins VP1 and VP3. Furthermore, as in TrV, the VP4 subunits are not structured in BQCV virions.

## RESULTS AND DISCUSSION

### Structure of BQCV virion and capsid proteins.

**FIG 1:**
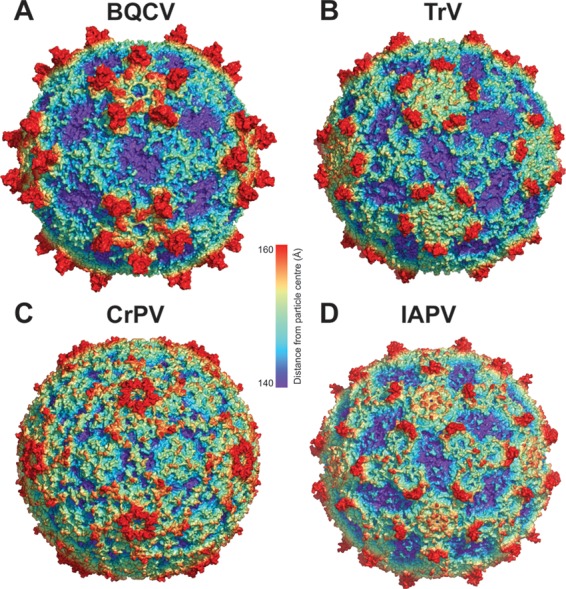
Comparison of virion structures of BQCV, TrV, CrPV, and IAPV. Molecular surfaces of BQCV (A), TrV (B), CrPV (C), and IAPV (D) virions are rainbow-colored based on their distance from the virion center. Depressions are shown in blue and protrusions in red.

**FIG 2:**
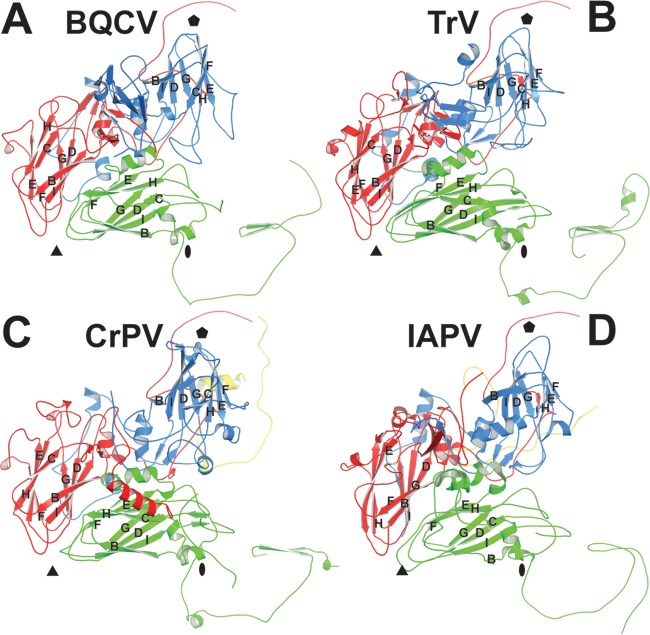
Comparison of structures of icosahedral asymmetric units of BQCV, TrV, CrPV, and IAPV. Shown are cartoon representations of the capsid protein protomers of BQCV (A), TrV (B), CrPV (C), and IAPV (D). VP1 subunits are shown in blue, VP2 in green, VP3 in red, and VP4 (if present) in yellow. Names of the β-strands of the capsid proteins are shown. The positions of the 5-fold, 3-fold, and 2-fold icosahedral symmetry axes are indicated with pentagons, triangles, and ovals, respectively.

The crystal structure of the BQCV virion was determined to a resolution of 3.4 Å (Table 1). The maximum outer diameter of the BQCV capsid is 353 Å (Fig. 1A). The particles of BQCV are bigger than those of other dicistroviruses and most picornaviruses (maximum radii of about of 320 Å) because of the finger-like protrusions located in between the 5-fold and 3-fold axes of the icosahedral symmetry of the BQCV capsid (Fig. 1). The virion has pseudo-T=3 icosahedral symmetry with 60 copies of each of the viral structural proteins VP1, VP2, and VP3. VP1 subunits form pentamers around the 5-fold axes, whereas VP2 and VP3 subunits constitute alternating heterohexamers around the icosahedral 3-fold axes (Fig. 2). The major capsid proteins have β-sandwich “jelly roll” folds. The β-strands forming the cores of the subunits are named according to the virus jelly roll convention B to I (25). The two antiparallel β-sheets contain strands BIDG and CHEF, respectively (Fig. 2A). The N termini of the major capsid proteins are located on the inside of the capsid, whereas the C termini are exposed at the virion surface. A complete model of the major capsid proteins of BQCV could be built except for seven C-terminal residues of VP3. BQCV encodes the 75-residue-long capsid protein VP4. However, no density corresponding to VP4 could be identified in the virion structure. The consequences of the missing VP4 structure for BQCV infectivity are discussed below.

**TABLE 1:** BQCV virion structure quality indicators

Parameter	Value^*a*^
Space group	I222
Cell parameters	
*a*, *b*, *c* (Å)	332.86, 350.60, 362.61
α, β, γ (**°**)	90.0, 90.0, 90.0
Resolution (Å)	40.00–3.40 (3.59–3.40)
*R*_merge_^*b*^	0.208 (0.633)
I/σ(I)	3.7 (1.0)
Completeness (%)	68.1 (63.3)
Multiplicity	1.9 (1.7)
No. of observations	359,305 (44,199)
No. of unique reflections	193,433 (26,184)
R_work_^*d*^	0.247
Avg atomic B factor (Å^2^)	46.4
RMSD bond angles (°)	0.848
RMSD bond lengths (Å)	0.007
Ramachandran statistics^*c*^	
Favored (%)	92.2
Outliers (%)	0.7
Molprobity score	9.75 (73rd percentile)

a Statistics for the highest-resolution shell are shown in parentheses.

b *R*_merge_ = Σ_h_Σ_j_|l_hj_−<l_h_>|/ΣΣ|l_hj_|.

c According to the criterion of Molprobity (76).

d All reflections were used in the refinement. The R_free_, if it were calculated, would be very similar to *R*_work_ because of the 15-fold noncrystallographic symmetry present in the crystal. Therefore, the *R*_free_ would not provide an unbiased measure of model quality in this case (71).

### Comparison of BQCV capsid structures to those of other dicistroviruses.

**FIG 3:**
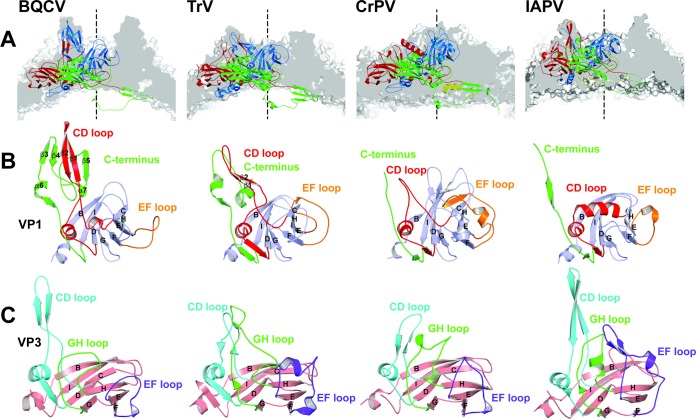
Comparison of prominent virion surface features of BQCV, TrV, CrPV, and IAPV. (A) Cross section of capsids close to 5-fold icosahedral axes are shown in gray. Cartoon representations of capsid proteins from a selected icosahedral asymmetric unit are shown in blue for VP1, green for VP2, red for VP3, and yellow for VP4. Finger-like protrusions of BQCV formed by the C terminus of VP1 and CD loops of VP1 and VP3 are larger than those of TrV, CrPV, and IAPV. The positions of the 5-fold icosahedral symmetry axes are indicated with dashed lines. (B) Comparison of VP1 subunits is shown. The CD loops are highlighted in red, the EF loops in orange, and the C termini in green. Names of the secondary structure elements are indicated. (C) Comparison of VP3 subunits. The CD loops of VP3 are highlighted in cyan, the GH loops in green, and the EF loops in magenta.

BQCV represents the first structurally characterized virus from the genus Cripavirus infecting honeybees. It shares less than 35% sequence identity with CrPV, TrV, and IAPV (Table 2) (21–23) and has a rather unique surface topology characterized by the large finger-like protrusions (Fig. 1A and 3A). There are plateaus around the icosahedral 3-fold axes and broad depressions on the BQCV virion surface around the icosahedral 2-fold axes (Fig. 1A). BQCV is structurally the closest to TrV, with a root mean square deviation (RMSD) of 1.9 Å for the Cα atoms of residues from icosahedral asymmetric units (Table 2) (23). The two viruses have similar surface features; however, the “fingers” of TrV are less prominent (Fig. 3A). In contrast, the virion surface of CrPV is almost flat (Fig. 3A) (22).

**TABLE 2:** Sequence and structural similarity of capsid proteins of selected dicistroviruses, iflaviruses, and picornaviruses

Family	Genus	Virus	RMSD (Å) of superimposed Ca atoms of the respective 3D structures (top) or % identity between the respective virus coat protein sequences (bottom)^*a*^																
			BQCV	TrV	CrPV	IAPV	PV1	CVB3	EV71	HRV16	FMDV	ERAV	TMEV	MEV	SVV1	AiV	HAV	HPeV-1	SBPV
Dicistroviridae	Cripavirus	BQCV		1.9	1.8	2.4	2.6	2.6	2.6	2.6	2.6	2.7	2.8	2.6	2.8	2.6	2.5	2.6	2.1
		TrV	33		1.9	2.0	2.3	2.3	2.3	2.4	2.8	2.5	2.5	2.4	2.7	2.5	2.2	2.4	2.0
		CrPV	29	29		2.1	2.6	2.5	2.7	2.5	2.7	3.0	2.8	2.7	3.2	2.6	2.6	2.7	2.2
	Aparavirus	IAPV	24	23	24		2.6	2.6	2.6	2.8	3.0	2.5	2.6	3.0	2.5	2.6	2.2	2.4	2.1
Picornaviridae	Enterovirus	PV1	14	16	13	11		1.0	1.1	1.0	1.7	1.8	1.5	1.5	2.3	2.6	2.1	2.1	2.3
		CVB3	14	16	13	11	56		1.1	0.9	1.9	1.8	1.6	1.6	1.9	1.9	2.1	2.4	2.5
		EV71	13	16	13	12	45	48		1.1	1.9	1.7	1.7	1.7	1.8	2.5	2.1	2.2	2.2
		HRV16	14	15	14	9	49	49	45		2.1	2.0	1.6	1.6	2.0	2.1	2.0	2.3	2.3
	Aphthovirus	FMDV	13	15	13	12	26	26	27	23		1.6	1.6	1.5	1.6	1.9	2.1	2.7	2.6
		ERAV	17	18	17	11	24	26	23	26	36		1.9	1.6	1.9	2.0	2.4	2.6	2.8
	Cardiovirus	TMEV	19	15	14	13	29	29	30	27	31	33		0.9	1.4	1.9	2.0	2.1	2.3
		MEV	18	16	15	12	29	29	30	30	30	35	65		1.3	1.7	2.1	2.1	2.3
	Senecavirus	SVV1	15	12	11	14	29	25	29	25	30	31	38	40		1.8	2.0	2.2	2.4
	Kobuvirus	AiV	16	14	16	16	24	21	26	24	19	25	28	27	28		2.1	2.2	2.3
	Hepatovirus	HAV	17	21	16	14	18	19	17	19	17	18	21	19	20	17		2.1	2.1
	Parechovirus	HPeV-1	16	16	17	15	17	13	17	11	17	18	20	21	18	20	18		2.2
Iflaviridae	Iflavirus	SBPV	17	21	19	19	16	15	14	14	15	16	16	16	14	21	18	17	

a For RMSD, the distance cutoff for inclusion of residues in the calculation was 3.8 Å. Capsid protein protomers corresponding to icosahedral asymmetric units consisting of subunits VP1 to VP4 were used in the comparisons. The program Coot was used for superposition of the molecules (69). For percent identity between the respective virus coat protein sequences, gaps were ignored in the calculations. Abbreviations: poliovirus 1 (PV1), coxsackievirus B3 (CVB3), human rhinovirus 16 (HRV16), foot-and-mouth disease virus (FMDV), equine rhinitis A virus (ERAV), Theiler's encephalomyelitis virus (TMEV), mouse encephalomyelitis virus (MEV), Seneca Valley virus (SVV1), Aichi virus (AiV), hepatitis A virus (HAV), and human parechovirus 1 (HPeV-1).

The finger-like protrusions of BQCV reach 34 Å above the virion surface (Fig. 1A and 3A). Each of the protrusions is formed by the C terminus of VP1 and CD loops of VP1 and VP3 (Fig. 3B). The C terminus of VP1 of BQCV is 21 residues longer than that of TrV (23). The 47-residue-long C terminus of BQCV VP1 contains α-helix 6 followed by β-strands 3, 4, and 5 and α-helix 7 (Fig. 3B). The CD loop of VP1 of BQCV is four residues longer than that of TrV. In BQCV the loop contains a four-residue-long α-helix 4 followed by β-strands 1 and 2 and an eight-residue-long α-helix 5 (Fig. 3B). The CD loop of VP3 of BQCV is seven residues longer than those of TrV and CrPV (Fig. 3C) (22, 23). The CD loop of VP3 of IAPV is similar in size to that of BQCV (21). The CD loop of BQCV VP3 contains three β-strands and an α-helix (Fig. 3C). The smaller finger-like protrusions of TrV and IAPV are formed by the C terminus and CD loop of VP1 but not by the CD loop of VP3 (Fig. 3B) (21, 23). There are no finger-like protrusions in CrPV (Fig. 3A) (22). Previously, the finger-like protrusions of TrV were speculated to play a role in the interactions of the virus with its host, in particular to be involved in binding to the entry receptor (23).

The EF loop of VP1 of BQCV is 13 residues shorter than that of CrPV, 2 residues shorter than that of TrV, but 5 residues longer than that of IAPV (Fig. 3B) (21–23). In BQCV the loop does not contain any secondary-structure elements. In contrast, the EF loop of CrPV VP1 contains an α-helix and β-strand (22). The most prominent surface feature formed by subunit VP2 of BQCV is the EF loop, which is, according to the picornavirus convention, named the “puff.” The puff regions of the dicistroviruses are similar (Fig. 2).

The GH loop of VP3 of BQCV is the shortest among the structurally characterized dicistroviruses and lacks the α-helix and β-strand that are present in the GH loops of TrV, CrPV, and IAPV (Fig. 3C) (21–23). The GH loop of VP3 in TrV is the longest of the compared viruses. In contrast, the GH loop of VP3 of CrPV contains two short α-helices (Fig. 3C). The EF loop of VP3 in BQCV is the shortest of all the compared viruses and contains only one short α-helix (Fig. 3C). The longest EF loop of VP3 can be found in IAPV, in which it is formed by two β-strands followed by an α-helix (Fig. 3C) (21).

**FIG 4:**
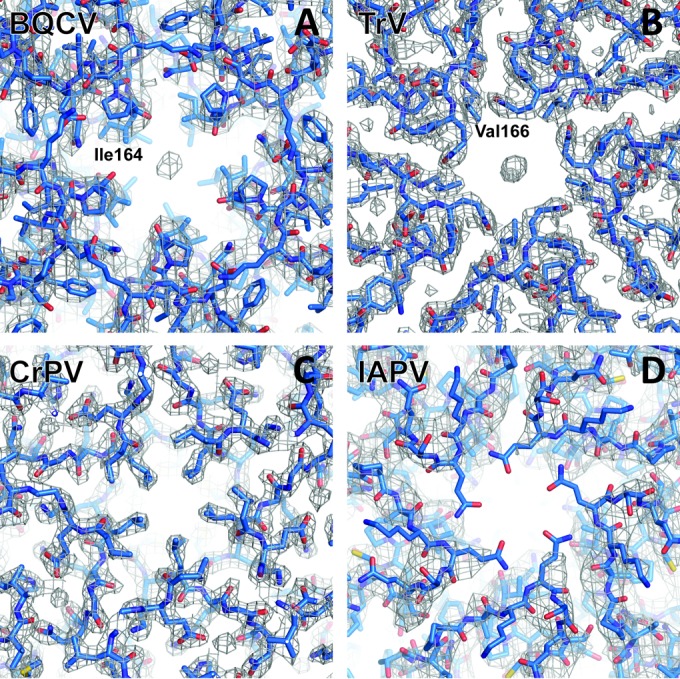
Maps of electron densities of capsids of dicistroviruses close to icosahedral 5-fold axes. Electron densities attributed to putative ions are present on 5-fold axes of BQCV (A) and TrV (B). In contrast, the density is absent in CrPV (C) and IAPV (D). The density maps are shown as gray meshes contoured at 1.8 σ. VP1 subunits are shown in stick representation with carbon atoms in blue. The names of residues of BQCV and TrV closest to the putative ion densities are shown.

The capsid of BQCV contains a spherical electron density positioned on a 5-fold axis in the vicinity of the Ile 164 residues of symmetry-related VP1 subunits (Fig. 4). A similar density has been previously observed in the capsid of TrV, where it was attributed to an ion (23). In contrast, no density was observed in the same region of virions of CrPV and IAPV (Fig. 4) (21, 22). It has been speculated previously that the ions may contribute to the capsid stability of viruses, and they might have similar functions in BQCV and TrV.

### The BQCV capsid lacks resolved density for minor capsid protein VP4.

**FIG 5:**
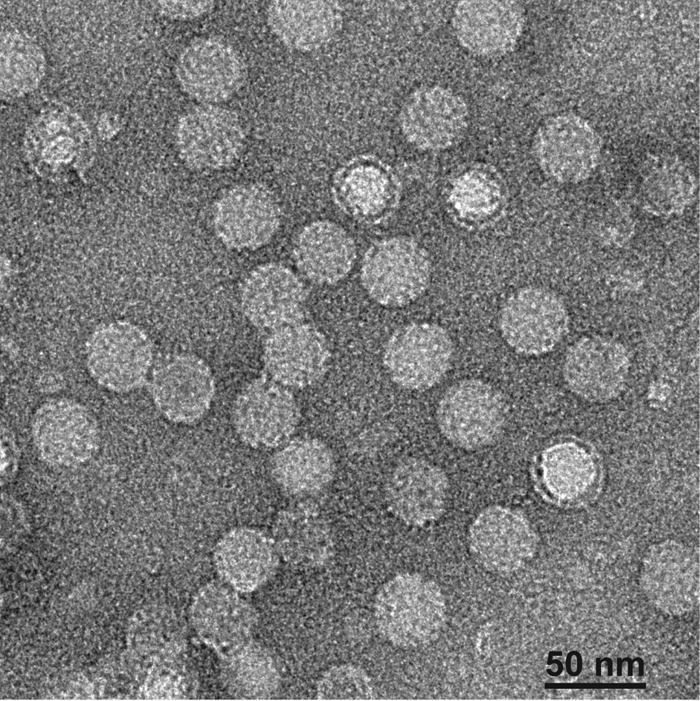
Negative-stain electron microscopy picture of BQCV after purification on CsCl gradient. See Materials and Methods for details on the purification procedure.

**FIG 6:**
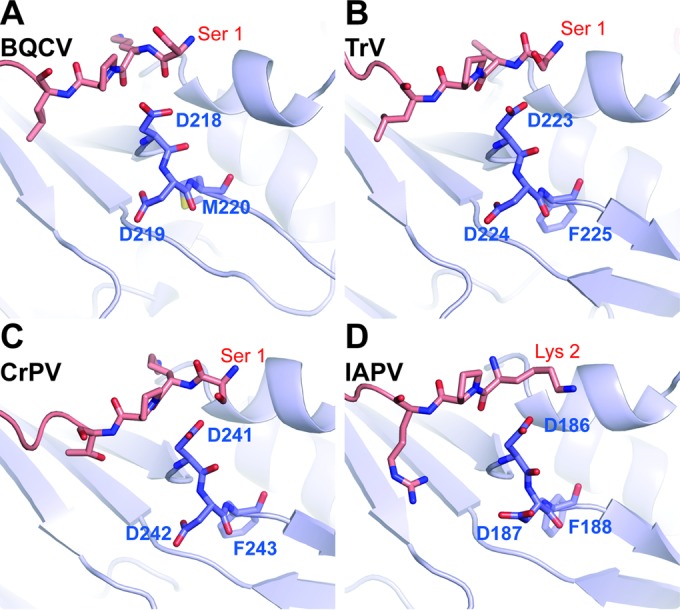
Putative proteolytic site in VP1 subunits of dicistroviruses. The residues Asp-Asp-Phe/Met of VP1 that were speculated to mediate the cleavage of VP0 into VP3 and VP4 are positioned close to the N terminus of VP3 and C terminus of VP4 from another protomer related by an icosahedral 5-fold axis of symmetry. The residues constituting the putative active site are shown in stick representation. VP1 subunits are shown in blue and VP3 in red.

Virions of many viruses from the order Picornavirales assemble as immature particles that contain the precursor subunit VP0 (39). Formation of the mature infectious virions is, in such cases, dependent on the cleavage of capsid protein VP4 from the N terminus of the VP0 precursor. In picornaviruses, the VP0 cleavage generates the proteins VP4 and VP2, whereas in dicistroviruses the precursor cleavage results in the formation of VP4 and VP3 (22, 23). Infections of some picornaviruses produce not only genome-containing virions but also empty particles that have VP0 subunits. However, the purification of BQCV in a CsCl density gradient resulted in the formation of one band, which contained only full virions (Fig. 5). It has been speculated previously that a conserved Asp-Asp-Phe (DDF) motif, which is part of the VP1 subunit, is involved in the VP0 cleavage of dicistroviruses (22, 23, 30). IAPV, CrPV, and TrV contain the DDF motif in a loop immediately following β-strand I of VP1 positioned on the inside of the capsid. Furthermore, TrV and IAPV have additional DDF sequences, in a loop following β-strand I of VP3 (22, 23). The VP1 subunit of BQCV contains an alternative sequence, DDM, at residues 218 to 220, located in a position similar to those of the DDF sequences of TrV, CrPV, and IAPV (Fig. 6). Cleavage of the VP0 precursor generates a new N terminus of VP3, which starts with Ser1 (Fig. 6A to C). With IAPV, the N-terminal serine was not resolved in the electron density map and the structure starts from Lys2 (Fig. 6D). Asp218 of the BQCV DDM motif is located close to the N terminus of VP3 (Fig. 6A). Their relative positioning indicates that the formation of pentamers is sufficient to achieve an optimal spatial arrangement of the putative autocatalytic center formed by residues of VP1 for the cleavage of VP0. The mechanism that ensures that the VP0 cleavage occurs only in dicistrovirus virions containing the RNA genome (22, 40) remains to be determined.

**FIG 7:**
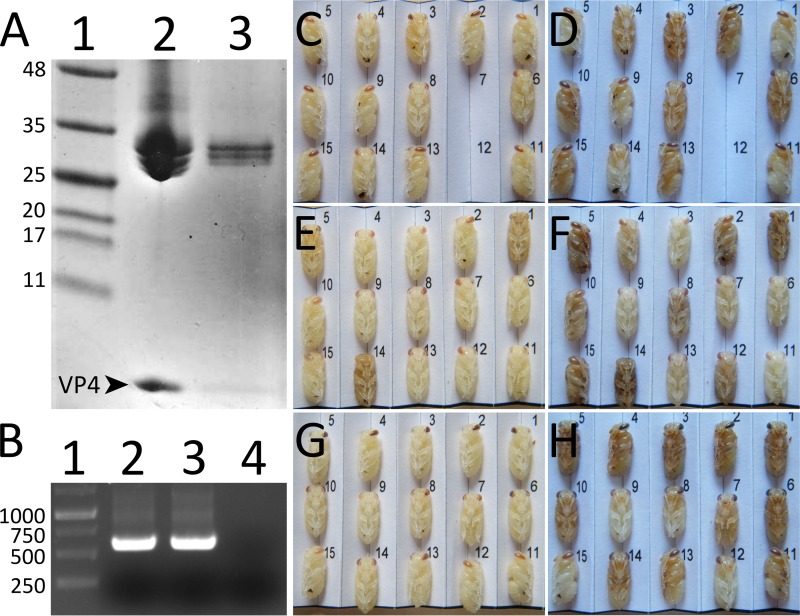
BQCV crystals contain VP4 subunits and the crystallized virus is infectious. (A) Polyacrylamide gel electrophoresis of capsid proteins of BQCV. Lane 1, marker; lane 2, purified BQCV; lane 3, BQCV dissolved from crystals. Arrowhead and VP4 label indicate the position of capsid protein VP4 (8.1 kDa). Capsid proteins VP1, VP2, and VP3 of BQCV have molecular masses in the 25- to 35-kDa range. (B) Agarose gel electrophoresis of PCR fragments obtained from reverse-transcribed RNA isolated from pupae injected with native BQCV (lane 2), BQCV dissolved from crystals (lane 3), and mock-infected with PBS (lane 4). Please see Materials and Methods for details. Lane 1, DNA ladder. (C to H) Images of pupae injected with BQCV dissolved from crystals (C and D) or native virus (E and F) or mock infected with PBS (G and H). The pupae were imaged 1 day (C, E, and G) and 5 days (D, F, and H) after the injection. The pupae injected with virus (C to F) developed slower than the mock-injected pupae (G and H), as shown by the delay in color development of the eyes and the darkening of the body 5 days postinfection. Two pupae missing in the panels (C and D) were accidentally destroyed during imaging.

As with BQCV, the previously determined structure of the TrV virion lacked a resolved electron density for the VP4 subunits (23). However, it was shown that TrV virions contain VP4 peptides and that dissolved TrV crystals could be used to infect triatoma insects. Therefore, VP4 peptides are unstructured components of TrV virions (23). In contrast, electron density maps enabled the VP4 structures in CrPV and IAPV to be built (21, 22). It was proposed that one characteristic of viruses from the genus Triatovirus within the family Dicistroviridae is the absence of structured VP4 subunits (23, 30). SDS gel electrophoresis and mass spectrometry analysis showed that VP4 subunits are present in both native and crystallized BQCV virions (Fig. 7A; see also Fig. S1 in the supplemental material). Furthermore, BQCV genomes could be detected in pupae injected both with the native virus and with particles dissolved from crystals (Fig. 7B). Honeybee pupae injected with BQCV dissolved from crystals stopped their development, similar to those injected with the native virus (Fig. 7C to H). The results show that BQCV virions are infectious even without the structured VP4 subunits, similar to what was shown for TrV (23). However, because the VP4 cleavage is probably required for BQCV maturation, it is likely that at least before maturation the residues corresponding to VP4 are ordered in the capsid. It is also possible that some of the crystallized virions could have lost VP4 by externalization in an aborted entry reaction during *in vitro* handling of the virus, leaving insufficient capsid-associated material to provide a resolved density for VP4.

### Absence of hydrophobic pocket in VP1 of BQCV.

**FIG 8:**
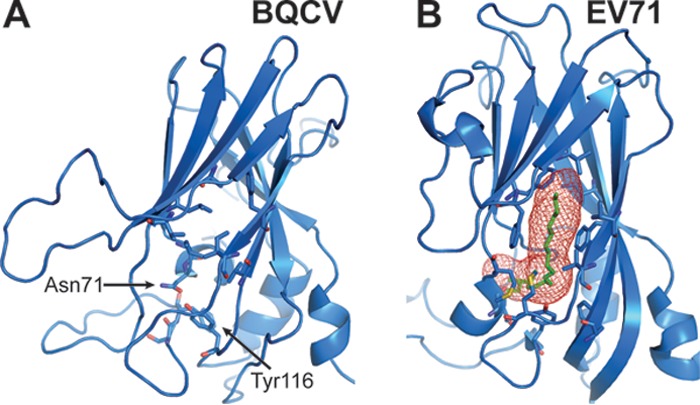
VP1 of BQCV does not contain a hydrophobic pocket. VP1 of BQCV (A) and human enterovirus 71 (EV71) (B) are shown in cartoon representations. The pocket factor of human enterovirus 71 is shown as a stick model in green. The volume of the pocket calculated with the program Caver is shown in panel B. In addition, the side chains of residues that interact with the pocket factor are shown as sticks. In BQCV, the core of the VP1 subunits is filled by side chains of residues forming the β-sheet BIDG and CHEF. The residues Asn71 and Tyr116 in BQCV obscure the volume that corresponds to the opening of the pocket at the capsid surface in EV71.

Dicistroviruses are related to vertebrate picornaviruses, for which numerous capsid-binding inhibitors have been developed (41). The VP1 subunits of some enteroviruses, including human enterovirus 71 (EV71), contain a hydrophobic pocket that can be targeted by small compounds which inhibit the virus-receptor binding and/or genome release (42–45). However, BQCV does not harbor such a hydrophobic pocket in the β-barrel of VP1 (Fig. 8A). The β-barrel of BQCV VP1 is compressed compared to that of EV71, and the remaining space is taken up by hydrophobic side chains of amino acids forming the core of the protein (Fig. 8). In addition, the residues Asn71 from β-strand C and Tyr116 from the CD loop of VP1 occupy the volume of the putative entrance to the pocket (Fig. 8A). Previous structural analyses of CrPV, TrV, and IAPV have shown that these viruses also lack pocket factors (21–23). Therefore, it is likely that pocket binding inhibitors may not be effective as antivirals against honeybee viruses from the family Dicistroviridae.

### Evolutionary relationship to dicistroviruses, iflaviruses, and picornaviruses.

**FIG 9:**
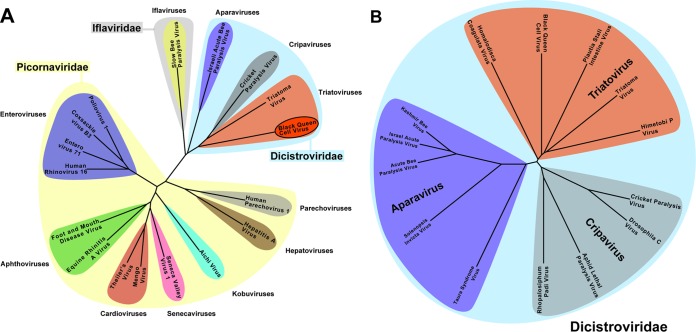
Evolutionary relationship among viruses from the Dicistroviridae, Picornaviridae, and Iflaviridae families based on structural alignment of capsid proteins. (A) Phylogenetic tree based on structural similarity of icosahedral asymmetric units of indicated viruses. (B) Evolutionary tree of dicistroviruses based on alignments of ORF2 sequences verifies division of dicistroviruses into genera Aparavirus, Cripavirus, and Triatovirus. For details on the construction of the diagram, please see Materials and Methods.

A structure-based evolutionary tree derived from a comparison of icosahedral asymmetric units clearly separates the families Dicistroviridae, Iflaviridae, and Picornaviridae (Fig. 9A). The structural comparison indicates that dicistroviruses are most similar to iflaviruses, which also infect insects (21–23). The viruses closest to BQCV from the Picornaviridae family are hepatitis A virus and human parechovirus 1, which were previously suggested to form evolutionary intermediates between human and insect viruses (Fig. 9A) (46, 47).

In order to expand our analysis to viruses with unknown structures, we calculated an evolutionary tree of viruses from the family Dicistroviridae based on the amino acid sequences of their ORF2 encoding the capsid proteins (Fig. 9B). The tree separates the viruses into three groups. One of them corresponds to the genus Aparavirus, which includes IAPV, acute bee paralysis virus (ABPV), Kashmir bee virus (KBV), taura syndrome virus, and Solenopsis invicta virus (48–52). Another genus is Cripavirus, structurally represented by CrPV and including Drosophila C virus, aphid lethal paralysis virus, and Rhopalosiphum padi virus (53–56). The remaining group is the recently formed genus Triatovirus, which is structurally represented by TrV and BQCV and also includes the Plautia stali intestine virus, Homalodisca coagulata virus, and himetobi P virus (Fig. 9B) (57–60). A difference that separates triatoviruses from cripaviruses, obvious only in the structural analysis, is the absence of ordered VP4 subunits in the virions of both BQCV and TrV. An additional distinction between cripaviruses and triatoviruses, which can be identified both in structures and in sequences, are the finger-like projections at the virion surface formed by the CD loop of VP1, which are present only in triatoviruses (Fig. 3B). Therefore, the structure of BQCV, which shares some of its unique features with TrV, reinforces the reasons for establishing the genus Triatovirus.

## MATERIALS AND METHODS

### Virus propagation in honeybee pupae.

The propagation of BQCV was carried out as described in the COLOSS BEEBOOK (61). Brood areas with Apis mellifera white-eyed pupae were identified by the color and structural features of the cell caps. White-eyed pupae were carefully extracted from the brood combs, so as not to injure the pupae. The pupae were placed on paper furrows with their ventral side up. In total 504 pupae were used for the BQCV propagation. The virus inoculum (1 μl) was injected into pupae with a Hamilton micropipette with a 30-gauge 22-mm-long needle through the intersegmental cuticle between the 4th and 5th sternites. Pupae that leaked hemolymph after the injection were discarded. The optimal concentration of the virus in the inoculum for virus production was determined experimentally, by comparing virus yields when using different virus concentrations in the injection inoculum. Inoculated pupae were placed into petri dishes with the paper furrows and incubated at 30°C and 75% humidity for 5 days. After incubation, the pupae were frozen at −20°C. For long-term storage, the pupae were kept at −80°C.

### Virus purification.

Fifty experimentally infected honeybee pupae were homogenized with a Dounce homogenizer in 30 ml of phosphate-buffered saline (PBS), pH 7.5 (Sigma-Aldrich). The nonionic detergent NP-40 was added to a final concentration of 0.5%, and the homogenate was incubated for 1 h at room temperature. The extract was centrifuged at 8,000 × *g* for 30 min. The pellet was discarded and the supernatant was centrifuged at 150,000 × *g* for 3 h in a Ti50.2 fixed-angle rotor (Beckman Coulter). The resulting pellet was resuspended in PBS to a final volume of 5 ml. MgCl_2_ was added to a final concentration of 5 mM; 20 μg/ml of DNase I and 20 μg/ml of RNase were added as well. The solution was incubated at room temperature for 30 min and centrifuged at 4,000 × *g* for 15 min. The resulting supernatant was loaded into a CsCl (0.6 g/ml) solution prepared in PBS. The ultracentrifugation at 220,000 × *g* proceeded for 16 h to establish the CsCl gradient. BQCV formed a single band in the CsCl gradient. The virus band was collected by gentle piercing of the ultracentrifuge tubes with an 18-gauge needle. The viruses were transferred to PBS by several rounds of concentration and dilution using centrifuge filter units with a 100-kDa molecular mass cutoff. This procedure yielded about 300 μg of virus with purity sufficient for screening. The nucleotide sequences of the virus preparations were determined by sequencing the RNA region encoding the capsid proteins. RNA was extracted from 10 infected honeybee pupae using TRIzol reagent. Viral RNA was reverse transcribed into cDNA using oligo(T) primers, which was used for commercial sequencing. The identical approach was used to prepare cDNA for detection of virus replication in pupae injected with BQCV from dissolved crystals. The primers used for subsequent PCR were 2F, with the sequence ACTCAAAGGATTTTCTTCTT, and 4R, with the sequence AAATAGGTCCTATGATTTCA. The resulting product was 599 bp in length.

### BQCV genome sequence and virus purity.

RNA was extracted from purified BQCV virions using a Qiagen RNEasy kit and the protocol for RNA cleanup. The RNA extracted from the BQCV virions was checked for the presence of other honeybee picorna-like viruses, a common problem of virus propagation in honeybee pupae (61), using previously reported virus-specific quantitative reverse transcription-PCR (RT-qPCR) assays for acute bee paralysis virus (ABPV), IAPV, KBV, deformed wing virus (DWV), BQCV, sacbrood virus (SBV), and slow bee paralysis virus (SBPV) (61). Only SBV and DWV were detected together with the purified BQCV virions. The total sum of SBV and DWV was less than 0.0001% (10^−6^) of the amount of BQCV. The full BQCV genomic sequence was determined by sequencing 300 ng of RNA using IonTorrent technology and standard protocols for library preparation and sequencing. The IonTorrent reads were mapped to the BQCV GenBank reference sequence (GenBank accession no. AF183905) using Tmap v4.4.8, included in TorrentSuite 4.4.2, with the parameters recommended by Life Technologies. Variability and consensus sequences were created using mpileup from samtools v.0.1.8 and an in-house script.

### BQCV crystallization and data collection.

The BQCV crystallization screening was performed at 20°C using the virus dissolved in PBS at a concentration of 3.4 mg/ml. Approximately 500 crystallization conditions were tested with the sitting-drop vapor diffusion method in 96-well plates. Initial conditions that produced crystals were optimized by using hanging drops in 24-well plates. Diamond-shaped crystals with a longest dimension of approximately 0.2 mm were obtained in 0.2 M ammonium acetate, 0.1 M bis-Tris (pH 7.5), and 35% 2-methyl-2,4-pentanediol (MPD). These crystals were flash frozen in liquid nitrogen without additional cryoprotectant and used to collect diffraction data at the PROXIMA-1 beamline of the Soleil synchrotron. The parameters used for data collection were as follows: crystal-to-detector distance, 623.7 mm; oscillation angle, 0.1°; exposure time, 0.1 s; X-ray wavelength, 0.97857 Å.

### BQCV structure determination and refinement.

BQCV diffraction data were indexed and integrated using the software package XDS (62). The BQCV crystal was of space group I222 (Table 1). Particle packing considerations indicated that the virus particle is positioned at the origin with a subset of icosahedral 2-fold axes aligned with the 222 symmetry axes of the crystal. Therefore, one-quarter of a virion occupied a crystallographic asymmetric unit. There were two alternative orientations of the icosahedral symmetry that could be superimposed with the 222 symmetry of the crystal. The orientation of the particle was determined from a plot of a 5-fold self-rotation function calculated using the program GLRF (63). Reflections with resolutions between 7 and 4 Å were used for the calculations. The radius of integration was set to 280 Å. The particle is rotated 90° about the Z-axis relative to the standard icosahedral orientation, as described by Rossmann and Blow (64).

The model of triatoma virus (TrV) (PDB entry 3nap) was used for the molecular replacement (23). The model was placed into the appropriate orientation and position in the unit cell and used to calculate phases to a resolution of 10 Å in CNS (65). The phases were refined by 25 cycles of averaging with the program AVE (66), using the 15-fold noncrystallographic symmetry. Other calculations, including map calculations from diffraction data and conversion of the averaged map into structure factor amplitudes and phases, were done using programs from the package CCP4 (67). The resulting map was used to recalculate the shape of the averaging mask based on a correlation map calculated using the program coma (68). Phase extension was applied in order to obtain phases for higher-resolution reflections according to the following procedure: the addition of a small fraction of higher-resolution data (one index at a time) was followed by three cycles of averaging. This procedure was repeated until phases were obtained for all the reflections to a resolution of 3.4 Å.

The structure was built manually from the TrV structure converted to polyalanine using the programs Coot and O and coordinate and B-factor refinement using the program CNS (65, 69, 70). Noncrystallographic symmetry constraints were applied during refinement. No water molecules were added to the crystal model due to the limited resolution of the diffraction data. All the measured reflections were used in the refinement. If calculated, the *R*_free_ value would be very similar to the *R* value, due to the 15-fold noncrystallographic symmetry present in the diffraction data (71).

### Structure and sequence analysis.

Multiple sequence alignments were carried out using ClustalW server (http://www.ebi.ac.uk/Tools/msa/clustalw2/) (72). Figures were generated using the programs UCSF Chimera (73) and PyMOL (PyMOL molecular graphics system, version 1.7.4; Schrödinger, LLC). Structure-based pairwise alignments of biological protomers of various picornaviruses were prepared using the program VMD (74). The similarity score provided by VMD was used as an evolutionary distance to construct a nexus-format matrix file, which was converted into the phylogenetic tree and visualized with the program SplitsTree (75).

### Mass spectrometry analysis.

The protein band corresponding to VP4 of BQCV was manually excised from SDS-PAGE gel. After destaining and washing, it was incubated with trypsin (sequencing grade; Promega). Matrix-assisted laser desorption ionization mass spectrometry (MALDI-MS) and tandem mass spectrometry (MS/MS) analyses of tryptic digests were performed on an Ultraflextreme mass spectrometer (Bruker Daltonics, Bremen, Germany). The FlexAnalysis 3.4 and MS BioTools 3.2 (Bruker Daltonics) software were used for data processing. Exported MS/MS spectra were searched with in-house Mascot (Matrixscience, London, UK; version 2.4.1) against the NCBI database (no taxonomy restriction) and a local database supplied with the expected sequence. Mass tolerances of peptides and MS/MS fragments for MS/MS ion searches were 50 ppm and 0.5 Da, respectively. Oxidation of methionine and propionylamidation of cysteine as optional modifications and one enzyme miscleavage were set for all searches. Peptides with a statistically significant peptide score (*P* < 0.05) were considered.

### Accession number(s).

Atomic coordinates of the BQCV virion at 3.4-Å resolution, together with the structure factors, were deposited in the Protein Data Bank under code 5MQC. The consensus nucleotide sequence of the BQCV capsid proteins and of the whole genome were deposited in GenBank under accession numbers KY363519 and KY243932, respectively.

## Supplementary Material

Supplemental material
