# Supplementary material for: Virion Structure of Black Queen Cell Virus, a Common Honeybee Pathogen
Source: J Virol. 2017 Feb 28;91(6):e02100-16. doi: 10.1128/JVI.02100-16 (PMC5331821; doi:10.1128/JVI.02100-16)
Supplement: Supplemental material [file supp_91_6_e02100-16__index.html]

Virion Structure of Black Queen Cell Virus, a Common Honeybee Pathogen — Supplemental material 

# Virion Structure of Black Queen Cell Virus, a Common Honeybee Pathogen

## Supplemental material

- Supplemental file 1 -

  Fig. S1 (Mass spectrometry analysis of proteins from BQCV showing that VP4 subunits are components of BQCV virions.)

  PDF, 1.0M
