# Supplementary material for: Virion Structure of Black Queen Cell Virus, a Common Honeybee Pathogen
Source: J Virol. 2017 Feb 28;91(6):e02100-16. doi: 10.1128/JVI.02100-16 (PMC5331821; doi:10.1128/JVI.02100-16)
Supplement: Supplemental material [file JVI.02100-16_zjv999182406s1.pdf]

A

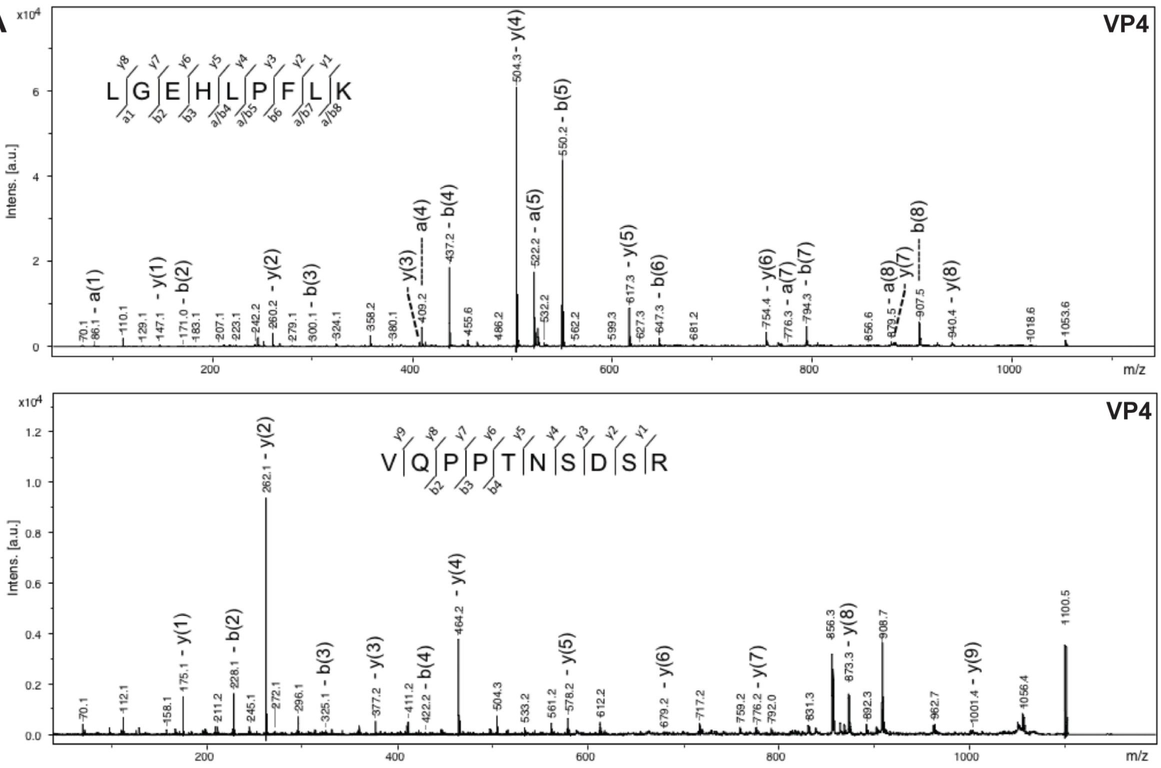

B

VP4

SGLK**VQPPTN SDSR**QRAKES KGKKDYSKTG AAFKQSVSK**L GEHLPFLK**SG ANWVSENILQ PADEIIGPIL SLFGF

Supplementary fig. 1. Mass spec analysis of proteins from BQCV showing that VP4 subunits are components of BQCV virions. Bands on SDS gel corresponding to VP4 shown in Fig. 7A were analyzed by Mass spectrometry. (A) Mass spectra of peptides from the putative VP4 sequences. (B) Amino acid sequence of VP4 of BQCV. Amino acids that are parts of the peptides that were detected in the Mass Spec analysis are highlighted in red.
